# Regression augmented weighting adjustment for indirect comparisons in health decision modelling

**DOI:** 10.1017/rsm.2025.10021

**Published:** 2025-07-10

**Authors:** Chengyang Gao, Anna Heath, Gianluca Baio

**Affiliations:** 1 Department of Statistical Science, University College London, London, UK; 2 Child Health Evaluative Sciences, Hospital for Sick Children, Toronto, ON, Canada; 3 Dalla Lana School of Public Health, University of Toronto, Toronto, ON, Canada

**Keywords:** indirect treatment comparisons, matching-adjusted indirect comparisons, parameteric G-computation, population adjustment

## Abstract

**Background:**

Understanding the relative costs and effectiveness of all competing interventions is crucial to informing health resource allocations. However, to receive regulatory approval for efficacy, novel pharmaceuticals are typically only compared against placebo or standard of care. The relative efficacy against the best alternative intervention relies on indirect comparisons of different interventions. When treatment effect modifiers are distributed differently across trials, population adjustment is necessary to ensure a fair comparison. Matching-Adjusted Indirect Comparisons (MAIC) is the most widely adopted weighting-based method for this purpose. Nevertheless, MAIC can exhibit instability under poor population overlap. Regression-based approaches to overcome this issue are heavily dependent on parametric assumptions.

**Methods:**

We introduce a novel method, ‘G-MAIC,’ which combines outcome regression and weighting-adjustment to address these limitations. Inspired by Bayesian survey inference, G-MAIC employs Bayesian bootstrap to propagate the uncertainty of population-adjusted estimates. We evaluate the performance of G-MAIC against standard non-adjusted methods, MAIC and Parametric G-computation, in a simulation study encompassing 18 scenarios with varying trial sample sizes, population overlaps, and covariate structures.

**Results:**

Under poor overlap and small sample sizes, MAIC can produce non-sensible variance estimations or increased bias compared to non-adjusted methods, depending on covariate structures in the two trials compared. G-MAIC mitigates this issue, achieving comparable performance to parametric G-computation with reduced reliance on parametric assumptions.

**Conclusion:**

G-MAIC presents a robust alternative to the widely adopted MAIC for population-adjusted indirect comparisons. The underlying framework is flexible such that it can accommodate advanced nonparametric outcome models and alternative weighting schemes.

## Highlights

**What is already known?**

- Health technology assessments often rely on indirect comparisons for evaluating treatment efficacy. Population adjustment is typically required to reduce the bias in these comparisons.
- When the trials used to construct the indirect comparison have small sample size and poorly overlapped population, the most popular population adjustment methods, Matching-adjusted indirect comparisons (MAIC) may produce non-sensible estimates without strong parametric assumptions.
- Regression-based adjustment like parameteric G-computation overcomes the problem of overlap, but relies heavily on parameteric assumptions

**What is new?**

- Inspired by Bayesian survey inference literature, we propose G-MAIC, a regression-based population adjustment method that occupies the middle ground between MAIC and parameteric G-computation.
- Using a comprehensive simulation study, we demonstrate that G-MAIC largely overcomes the instability problem of MAIC under small sample size and poor overlap.
- We show that population covariate structure can be important to the performance of population adjustment: joint population covariate distribution with non-linear dependency structure can break the implicit assignment model in MAIC, making it potentially more biased than non-adjusted methods.

**Potential impact for RSM readers**

- The proposed G-MAIC presents a robust and flexible alternative to the best parametric counterpart, with the potential to accommodate non-parametric regression models and different weighting approaches.
- Population covariate structure is another important factor to consider when evaluating the performance of population adjustment methods. Focusing solely on standard parametric distributions with different locations may obscure important limitations of these methods.

## 1 Introduction

Health technology assessment (HTA) plays a vital role in informing healthcare resource allocation decisions in publicly funded health systems, such as those in the United Kingdom, Canada, and Australia. Through systematic evaluation of the clinical and cost-effectiveness of health technologies, including drugs, devices, and procedures,^1^ ^,^ ^2^ HTA provides evidence-based recommendations to help decision-makers maximise health improvements within limited financial resources.

Randomised controlled trials (RCT) form the bedrock of clinical development and are required to demonstrate safety and efficacy for market approval.^3^ ^,^ ^4^ However, for novel interventions, reimbursement decisions are often made without direct RCT evidence on the relevant head-to-head comparisons (e.g., new vs existing drugs, rather than placebo).

In HTA, methods for ‘indirect’ comparisons estimate the relevant incremental clinical benefits even in the absence of RCTs directly comparing the two competing interventions. Standard indirect comparisons mainly focus on ‘connected’ networks, where two alternative interventions are assessed in two RCTs against a common comparator (often placebo or standard of care). In this case, the effect of the two interventions can be obtained by contrasting their relative effects against the common comparator. This is known as ‘Bucher’s method’^5^ and preserves randomisation within trial to provide an unbiased estimate of the treatment effect, under the assumption that it is constant across trials.^6^

Unfortunately, Bucher’s method is biased when effect-modifiers exist and are distributed differentially in the two trials, a common situation in practice. Thus, to provide accurate inference, analysts have to first ‘transport’ the relative treatment effects to a common population and then apply Bucher’s method. In a two-trial network with individual participant data (IPD) available from both trial populations, one could re-weight the IPD from one of the trials by the inverse odds of assignment to that study,^7^ which can balance the distribution of effect-modifiers across trials. However, due to confidentiality and commercial reasons, IPD are rarely available for all relevant sources. It is more common to have IPD from one company’s own sponsored trial, but only aggregated-level data (ALD), usually from summary statistics reported in published studies, for the comparator’s trial. The limited data accessibility poses additional challenges and has received an increasing amount of attention in recent years.^8^ ^–^ ^10^

When only ALD are available from one study in a two-trial network, population adjustment methods are either classified as ‘weighting-based’ or ‘regression-based.’ The most popular weighting-based method is Matching-Adjusted Indirect Comparisons (MAIC),^11^ ^,^ ^12^ while the most common regression-based methods are Simulated Treatment Comparisons (STC)^13^ and parametric G-computation.^14^ Recently, another method has been specifically designed for evidence synthesis in larger networks and is known as Multi-Level Network Meta Regression (ML-NMR).^15^ As a network evidence synthesis method, ML-NMR estimates a full outcome regression model by jointly synthesising data from multiple IPD and ALD studies. The resulting model naturally accommodates conditional estimands, and can be further marginalised over any chosen target population to produce the marginal estimand of interest—a key advantage for informing population-level decision-making.^15^ ^–^ ^17^

In the broader context of HTA, multiple relevant studies frequently exist beyond the simplified two-trial network we mentioned above, and methods like MAIC and STC are inherently limited to indirect comparisons of two trials. Consequently, such methods cannot coherently synthesise multiple sources of evidence across complex networks due to target population mismatch. Nevertheless, two-trial networks remain highly relevant, particularly when evidence is limited—such as shortly after regulatory approval when a novel treatment is only compared against placebo or standard care in an efficacy trial. Under such circumstances, indirect comparisons based on two-trial networks may offer critical preliminary evidence regarding comparative effectiveness while broader comparative evidence is still developing.

MAIC is a weighting-based method that generates weights based on the odds of assignment to the ALD trial in a limited data context. The weights, i.e., the odds of being assigned to the ALD trial, are not directly estimable due to the lack of IPD, but can be obtained through optimisation if combined with a moment-matching constraint.^11^ The optimised weights are used to fit a weighted regression model on the available IPD, with a treatment indicator as the only covariate. The estimated coefficient from this model quantifies the marginal treatment effect that might have been observed in the ALD population. MAIC provides unbiased estimate of the indirect treatment effect under the correct specification of the trial assignment model.^18^ ^,^ ^19^ However, poor overlap of the covariates combined with small sample sizes, a common issue in practice, can lead to extreme weights, potentially leading to higher bias than Bucher’s method.^20^ The associated bootstrap variance estimator^21^ can also be unstable, with weight estimation failing in some bootstrapped datasets and the estimated variance being unrealistically large.^22^

Regression-based methods can overcome the problem of unstable weights under poor population overlap by extrapolating beyond the covariate space of the IPD. A key consideration in using regression-based methods is the choice between marginal and conditional estimands; the former represents the average treatment effect averaged over a certain population, while the latter is conditional on specific covariate profile. As an example, the Simulated Trial Comparison (STC),^23^ which ‘reads off’ the regression coefficients as the estimate of population-adjusted treatment effects, typically targets the conditional estimand.^18^ ^,^ ^24^ This would not align with the marginal estimand for non-collapsible outcome measures, such as odds ratios and hazard ratios.^24^ ^,^ ^25^ We acknowledge that the selection of an estimand is critical but also nuanced, with profound implications on the transportability of the results, and there are varying perspectives regarding this choice.^24^ ^,^ ^26^ This manuscript will primarily focus on methods target the same estimand as used in MAIC, i.e., the marginal estimand, setting aside the broader debate for a more focused discussion.

Another regression-based adjustment method, Parametric G-computation, provides a marginal estimand even for non-collapsible outcome measures.^24^ ^,^ ^26^ ^,^ ^27^ It predicts the treatment effect in the ALD population by fitting an outcome regression model using IPD and extrapolating the fitted relationship to the simulated ALD population. However, this method assumes the covariates in the ALD population follow specific parametric distributions. The covariate-outcome relationship must also be correctly specified beyond the boundary of IPD, making analysts cautious about trusting results based on extrapolation and a fully simulated population.

We propose a method that occupies the middle ground between MAIC and parametric G-computation. This regression-based method requires a parametric outcome model but does not parametrise the covariate distribution in the ALD population. Instead, the ALD population is approximated by re-weighting the IPD population using mean-balancing weights, similar to MAIC. To coherently propagate uncertainty, we also adopt a Bayesian framework. The variance of the adjusted treatment effects can be estimated directly from the predicted treatment effects in each draw of approximated ALD population, operationalised via Bayesian bootstrap.^28^ This article introduces the population adjustment problem and discusses two main methods. We then introduce our proposed method before benchmarking it against existing methods in a simulation study. The simulation results are discussed, along with general issues in population adjustment, in the final section.

## 2 Overview of population adjustment

**Figure 1:**
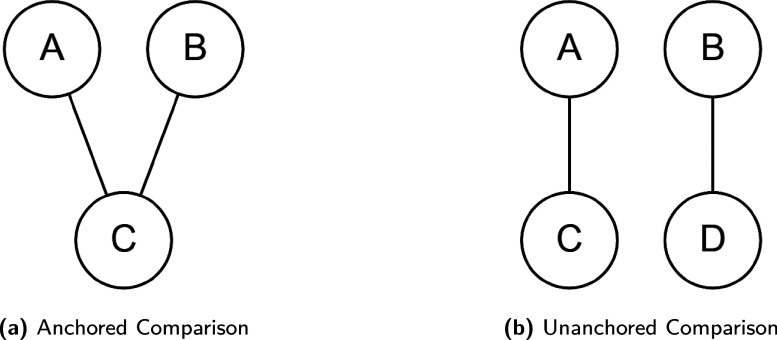
(a) shows an example of ‘Anchored comparison’, where the two treatments of interest *A* and *B* are compared against the common comparator *C* in two separate trials; (b) shows an example of ‘Unanchored comparison’ where the two treatments of interest *A* and *B* are compared against different comparators *C* and *D* in respective trials.

We aim to estimate the incremental benefit of intervention *A* compared to intervention *B*, to inform a decision model. We have evidence from two randomised trials, one for *A* and one for *B*, which may or may not have the same comparator to form a connected or disconnected ‘network’ (Figure 1a and 1b, respectively). An anchored network occurs when treatments *A* and *B* are connected through a common treatment *C*, allowing for unbiased indirect comparisons if the relative treatment effect remains constant. Conversely, the unanchored network (Figure 1b) requires stronger assumptions: the *A* vs *B* effect is unbiased only if the absolute effect of treatment *A* is constant, which requires the two trial population to be exchangeable in terms of both effect modifiers and prognostic factors. Given that this requirement can be too stringent to be realistic, we will focus on anchored comparisons.

For all discussions concerning anchored comparisons, we use the following notation for clarity: 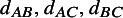 refer to the *A* vs *B*, *A* vs *C* and *B* vs *C* treatment effects respectively. The standard ‘Bucher’s method’ estimates 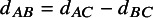 . As mentioned, this gives biased estimates in the presence of effect-modifiers. Population adjustment methods are thus required for a ‘fair comparison.’

### 2.1 MAIC

MAIC is likelihood reweighting applied in the limited data context.^29^ ^,^ ^30^ It determines the estimated treatment effect in the ALD population by reweighting the individual-level likelihood contributions in the IPD according to trial assignment odds, effectively estimating the treatment effect in a pseudo population that is exchangeable with the ALD. The calculation of weights consists of two pillars: a parametric model for the probability that each patient is enrolled in the ALD trial and a constraint that ensures equal means of effect modifiers across both trials. When MAIC was introduced,^11^ the weights were trial assignment odds, parametrised using their corresponding *K* effect-modifiers 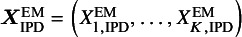 , on the log scale. In other words, if 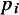 is the probability of individual *i* being assigned to the ALD trial, then the (log)-weights are computed as 
(1)

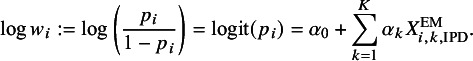



When we do not have access to individual-level data for the ALD population, a complete likelihood function cannot be formed and the coefficients in Equation 1 cannot be estimated using standard statistical methods.

Nevertheless, if we combine the expression for the 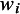 ’s with a mean-balancing constraint: 
(2)

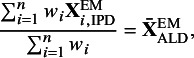

where 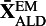 is the mean of the effect-modifiers in the ALD population, Equation (2) can be solved as an optimisation problem. The optimised weights are then used in a weighted regression with treatment indicator as the only covariate, equivalent to applying an unadjusted regression model to a population where the distribution of effect-modifiers are balanced, on average.

To quantify uncertainty in the MAIC estimator, we can use non-parametric bootstrap, which repeatedly samples the IPD to compute the weights and run a weighted regression for each re-sampled dataset. This generates a ‘bootstrapped distribution’ of the MAIC estimates, whose empirical variance can be used as the estimate for variance of the MAIC estimator.

MAIC is appealing as it only assumes a logistic regression model for trial assignment. The correctness of this assignment model rest on the assumptions that the trial assignment odds are log-linear in covariates.^19^ ^,^ ^31^ From a population density estimation perspective, reweighting the IPD by MAIC weights lead to an exponential tilted population distribution. Implicitly, this implies that the assignment model would be mis-specified if the joint distributions of effect-modifiers are complex, or exhibit non-linear dependence structure. Additionally, the mean-balancing requirement in equation (2) enhances robustness; the final population-adjusted estimate will be unbiased irrespective of the trial assignment model, if the outcome model is linear in the covariates on the natural scale.^19^ ^,^ ^31^ However, popular models in HTA analysis, e.g., logistic regressions and survival models, are not linear in the covariates; thus, the accuracy of MAIC requires a correct trial assignment model. Standard MAIC estimates would be biased if the joint distributions of effect-modifiers within the ALD trial populations become complex, an example would be mixing in skewed or heavy-tailed distributions.^32^

### 2.2 Parametric G-computation

Regression-based methods view population adjustment as a prediction problem. In order to target a marginal treatment effect similar to MAIC, the marginal distribution of the outcome variable must be modelled in the target population with and without treatment.^14^Parametric G-computation uses IPD for the AC trial to fit an outcome model, which predicts the outcomes under different treatment conditions and computes the treatment effects of interest in the fully-parametrised ALD trial population.

Although parametric G-computation has no theoretical restrictions on the type of outcome, here we illustrate it for a binary outcome 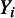 , where the probability of occurrence is described for each individual 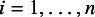 as 
(3)

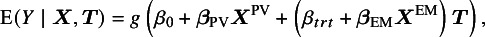

where 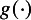 is the inverse logit function and the term 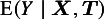 is the probability that the outcome occurs conditional on the treatment assignment 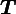 and a given covariates profile. The model includes: an intercept 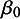 for the baseline odds; a coefficient 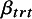 for the treatment effect that does not vary according to covariates; a vector 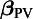 for the effect of the prognostic variables 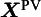 ; and a vector 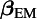 for the effect of the effect-modifiers 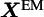 .

Remiro-Azocar et al. implemented this outcome regression in a Bayesian framework.^24^ After fitting the regression model, parametric G-computation is based on the distribution of effect-modifiers and prognostic variables in a target population. If we set 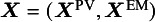 to include both prognostic variables and effect-modifiers, we then define the *theoretical* covariates distribution in the target population as 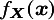 . Conditional on coefficients 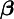 , individual-level predictions are aggregated to obtain the distribution of averages of hypothetical outcomes: 
(4)

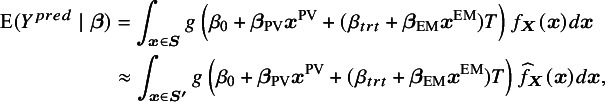

where 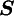 is the support of the theoretical distribution 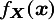 , which is generally unknown in practice. Thus, we approximate it using a finite population approximation 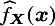 , defined over its support 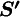 .

The posterior distribution of the conditional mean under either treatment condition, 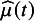 , can be found using a simulation approach by incorporating *L* posterior draws of the regression coefficients 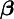 into Equation 4. Thus, the *l*-th draw of the average hypothetical outcomes can be calculated as 
(5)

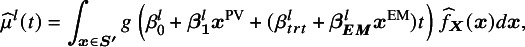

where *t* is the treatment condition of interest.

In standard G-computation, 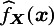 is usually the empirical covariate distribution of the sample IPD. However, in population adjustment, 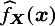 is based on simulating 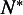 covariate profiles from the assumed ALD trial population, 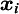 , 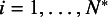 . Thus, (5) can be simplified to 

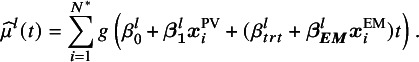

Finally, the *l*th draw from the posterior of the treatment effect can be computed as 

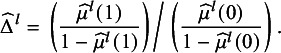



By directly parametrising the ALD population and extrapolating the outcome model beyond the covariate space of the IPD trial, parametric G-computation avoids the problem of unstable weights caused by the limited population overlap. In simulation studies, it provides more efficient estimates with minimal bias.^14^ However, parametric G-computation achieves this at the cost of stronger assumptions; it relies heavily on parametric assumptions—both the parametric representation of the ALD population and the outcome model must be correct for unbiased estimation. While the effect of model misspecification is still under investigation, results based on extrapolation and a fully simulated population may not be taken at face value.

### 3 G-MAIC

To overcome the limitations of the two procedures shown above, we propose a method, called G-MAIC, that reduces the small-sample bias and improves precision compared to MAIC, without explicitly parametrising the ALD trial population. G-MAIC combines weighting adjustment with G-computation and uses a variance estimator based on Bayesian bootstrap.^28^ As we will demonstrate in the simulation study in Section 4, it offers a promising alternative to MAIC, performing comparably to parametric G-computation.

Table 1 shows the connections between G-MAIC, MAIC, and parametric G-computation. G-MAIC requires a parametric outcome model, similar to parametric G-computation. However, instead of directly parametrising the distribution of effect-modifiers, G-MAIC indirectly parametrises 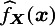 in Equation (5) by re-weighing the IPD using MAIC weights^1^, such that it resembles a population that is exchangeable to the ALD population. Similar to MAIC, this approach creates a pseudo population where the distribution of effect modifiers is, on average, balanced. The only difference being that G-MAIC reweighs the covariate instead of individual likelihood contribution as in MAIC.

**Table 1:** General building blocks for population adjustment methods targeting the marginal treatment effects

Method	Outcome model	Covariate model
MAIC	Implicit	Indirect
G-MAIC	Parametric	Indirect
Parametric G-computation	Parametric	Parametric

*Note*: ‘Implicit’ outcome model refers to the mean-matching step in MAIC guarantees its unbiasedness when the outcome is linear in covariates; ‘Indirect’ covariate model refers to first specifying a trial assignment model for weights estimation, and then use weights for approximating the ALD trial.

In order to propagate uncertainty from the approximated distribution of effect-modifiers in the ALD population to the estimated treatment effect, we propose a variance estimator based on Bayesian bootstrap.^28^ This variance estimator is more stable than the standard bootstrap used in MAIC, which can reduce the covariate space and result in extreme weights in poorly overlapped scenarios. As explained in the next section, the Bayesian bootstrap estimator treats approximating the effect-modifier distribution in the ALD population as a prediction task based on limited data, and considers the predictive distribution of the re-weighed population.

#### 3.1 Covariate approximation using Bayesian bootstrap

In our view, the primary challenge in population adjustment lies in accurately approximating the distribution of effect-modifiers, while adequately quantifying uncertainty. This is because explicit parametric assumptions must be made (which may be hard to justify) and, in addition, the uncertainty in the population approximation is often overlooked, as only a single draw from the assumed parametric distribution is utilised for the analysis.

We attempt to address these issues in the following manner. Consider the following approximation of 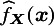 in (5): 
(6)

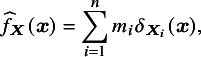

where 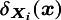 indicates that the effects modifiers are set to a fixed individual ‘profile’ 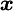 , without uncertainty, effectively assuming a degenerate distribution for 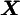 . The values 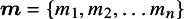 indicate weight parameters with the additional constraint that 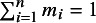 . By assigning different weights to different values of the effect modifiers, (6) can be employed to represent the empirical distribution of effect-modifiers in a finite sample. Additionally, if 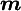 is drawn from a probability distribution, the uncertainty in 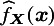 can be directly modelled.

Under a Bayesian framework, if the IPD and the ALD population can be seen as exchangeable, we can construct an approximation to 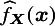 based on the empirical distribution of effect-modifiers in the IPD trial. Assume 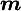 is drawn from an improper Dirichlet prior 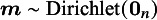 and suppose each observation corresponds to a unique covariate profile. The empirical data can be considered as a realisation of a Multinomial distribution with parameters equal to 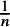 for all *n* observed values. Combining these data with the improper prior yields the conjugate posterior 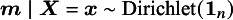 .

As explained by Oganisian et al.,^33^ this is the Bayesian bootstrap developed by Rubin.^28^ More intuitive interpretations of this procedure can be found in Bayesian survey inference under the term ‘Polya Sampling,’^34^ ^,^ ^35^ where the procedure is applied to impute the upsampled population based on sampled units. Bayesian bootstrap can also be conceptualised as ‘predictive resampling’ in Bayesian predictive inference, updating the ‘one-step predictive distribution’ by repeatedly resampling the Bayesian posterior.^36^ Both ideas show that, under exchangeability assumptions, Bayesian bootstrap is equivalent to modelling predictive distributions conditional on the known population.

In population adjustment, the two trial populations are not exchangeable. However, the re-weighed IPD based on MAIC weights can be viewed as being exchangeable to the ALD population, to a reasonable degree. By applying Bayesian bootstrap to the re-weighed IPD, we are essentially imputing the ALD population based on the pseudo population, with uncertainty of approximating the ALD contained within the predictive distributions.

#### 3.2 Statistical implementations

As stated in Table 1, G-MAIC assumes a parametric regression model similar to G-computation. In the marginalisation step, however, instead of averaging over a single simulated population, marginalisation is combined with Bayesian bootstrap to compute the average treatment effect with its associated uncertainty.

We have shown that applying Bayesian bootstrap to the re-weighted IPD gives us the posterior predictive distribution of the adjusted population. Therefore, each draw of the population-adjusted average outcomes under either treatment condition can be calculated as a weighted average by combining one posterior draw of the conditional mean with a set of random Dirichlet weights. The proposed population-adjusted treatment effect estimation can be implemented as follows.

We start by computing the normalised MAIC weights 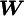 based on (1) and (2), where 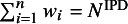 , using the full sample. This then serves as the basis to construct our random weight vector 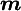 ; specifically, we replace the frequency of 1 in standard Bayesian bootstrap with the pseudo frequency 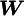 . Starting with the same non-informative prior for random weights, by the same Dirichlet-Multinomial conjugacy, the posterior distribution for the random weights 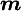 is: 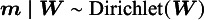 , the random Dirichlet weights can then be attached to the observations as in Equation (6).

Thus, the new adjusted distribution can be represented as 

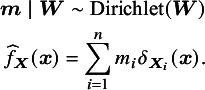



After fitting the same outcome regression model as in parametric G-computation, the full posterior inference for the treatment effect proceeds as in standard Bayesian G-computation. For the *l*th draw from the posterior distribution of 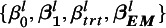 , a weight vector 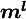 is drawn: 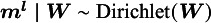 . The causal contrast can be computed as follows:

- For both *T* = {0,1}, average the conditional mean over the specific distribution of effect-modifiers: 

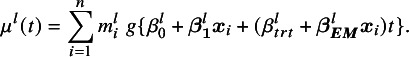

- Compute the causal contrast (e.g., the odds ratio): 

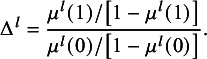

Repeating this step for 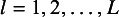 posterior draws gives *L* draws for the population-adjusted causal odds ratio, which could then be used to compute any summary of interest.

#### 3.3 Hypotheses and remarks

The proposed G-MAIC method employs the same logic as standard MAIC, which aims to estimate the treatment effect in a pseudo population with balanced effect-modifiers. However, the two methods differ in point and variance estimation. Instead of using an unadjusted estimator, G-MAIC utilises a ‘regression-then-marginalisation’ approach, which can increase efficiency, particularly for small sample sizes. The approach, coupled with Bayesian bootstrap that relies on MAIC weights calculated using the full sample, makes G-MAIC more reliable than traditional MAIC, as we will demonstrate in Section 5.

However, G-MAIC is still sensitive to population overlap. Limited population overlap leads to weight concentration, resulting in an underestimation of the variance of treatment effects. This problem becomes more severe as the overlap decreases. Therefore, when population overlap is poor, parametric G-computation is preferred due to its ability to extrapolate. Based on the above features of G-MAIC, we undertook the simulation study to verify the following hypotheses:

1. G-MAIC will have smaller variance than MAIC in small samples.
2. G-MAIC will have a more stable variance estimator than MAIC.
3. G-MAIC will have incorrect nominal coverage when overlap is poor.
4. G-MAIC will have worse under-coverage for small effective sample sizes (ESS).
5. G-MAIC will provide worse estimates than parametric G-computation with poor overlap.

### 4 Simulation studies

#### 4.1 Aim

This simulation study aims to examine whether G-MAIC can improve over standard MAIC in challenging anchored two-trial scenarios. We also aim to benchmark the performance of G-MAIC against parametric G-computation, which is not affected by poor trial population overlap. We expect G-MAIC to offer efficiency gains while being relatively robust when the trial assignment model of MAIC is mis-specified. We will evaluate the following metrics under different data-generating mechanisms: (1) unbiasedness; (2) variance unbiasedness; (3) coverage; (4) precision. The simulation study is reported following the ADEMP (Aim, Data-generating mechanisms; Estimands; Methods; performance measures) structure.^37^ The simulation study was implemented in R software version 4.1.1,^38^ with R code available on github at https://github.com/garoc371/G-MAIC/tree/main.

#### 4.2 Data-generating mechanisms

We consider a binary outcome, generated under a logit model as in (3). The two trials are 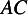 and 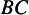 , involving three treatment 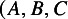 ) with *C* being the common comparator.

We first generate IPD for both trials, with the 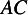 trial designated as the IPD trial. The IPD consist of individual-level outcomes 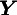 , the treatment assignment status 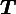 and a matrix of five covariates 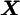 . All covariates are assumed to be prognostic variables and effect-modifiers, i.e., 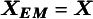 in equation (3). The *BC* trial is the ALD trial so the simulated IPD are aggregated to obtain covariate summaries; overall event counts and the number of participants by treatment, where, specifically 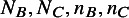 are the sample sizes and overall event count in arm *B* and *C*. We fix the size of the ALD trial at 600, corresponding to a typical Phase III trial,^39^ with a 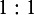 allocation ratio.

The model for the probability of experiencing the outcome is: 

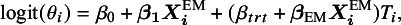

where 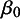 is the fixed control group event rate. The strength of effect-modification 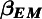 is assumed to be the same under the assumption of shared effect-modifiers,^17^ and we set 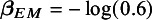 , corresponding to strong effect-modification. The prognostic strength 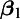 is also assumed to be the same and set to 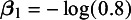 . The treatment effects are assumed to be equally large in both trials with 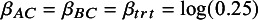 . The treatment effects correspond a decrease in baseline odds by 75% but with strong effect-modification, the sign of the treatment can still be flipped.

The estimand of interest is the *A* vs *B* marginal treatment effect in the BC population. Based on the current setup, the true *A* vs *B* effect is zero.

To assess the performance of population adjustment methods, we set the IPD sizes at 100, 200, or 600. We devised high, moderate, and low population overlaps by adjusting covariate distributions, each corresponding to different degrees of reductions in ESS. Factorial combinations of these factors in multivariate normal and non-normal covariate structures yield 18 scenarios. Detailed parameter configurations are in the appendix.

#### 4.3 Methods

The following methods will be compared:

1. Bucher’s method
2. MAIC
3. Bayesian parametric G-computation from Remiro et al.^14^
4. Bayesian regression marginalisation with MAIC weights
5. Bayesian parametric G-computation with misspecified covariate structure:
  - With the true Normal covariate structure, the ALD population is generated from a multivariate Gamma distribution
  - With the true non-normal covariate structure, a multivariate normal distribution is used to generate the ALD population.

STC and ML-NMR^15^ are excluded in the simulation study. This is because the STC targets the conditional estimand while we are targeting the population-adjusted marginal effect. Conversely, the operation of ML-NMR, is similar to STC in two-trial targeted comparison scenario.^22^ It can target the marginal estimand by parametrising the ALD population and marginalising over it, which is equivalent to the parametric G-computation.

#### 4.4 Performance measures

We simulate 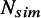 datasets for each scenario and estimate the treatment effect ( 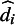 ) of A vs B in the BC population. The true value of this effect (*d*) is zero. The bias of the population-adjusted estimator is the expected difference between the estimated value and the truth. Variability is measured using empirical standard errors, (the standard deviation of 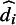 across all repetitions), model average standard errors (the average of the standard error associated providing by the estimating procedure 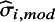 ). If these two measures are close, this reflects that the variance estimator is stable. Finally, coverage is the proportion of 95% confidence intervals that contain the true difference using Wald-type confidence intervals with 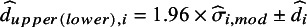 . These measures can be calculated as:

- Bias 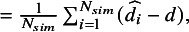

- Empirical standard error 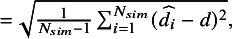

- Model average standard error 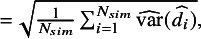

- Coverage 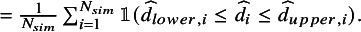

To determine 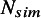 , the Monte Carlo standard errors (MCSE) of the performance measures should be low relative to the estimates. Since the bias and precision are of primary interests, we base the number of repetitions on the MCSE of them: 

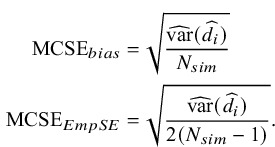

We consider 2,000 Monte Carlo replications for our analysis. And we conduct a further simulation with 5,000 Monte Carlo replications to ensure stability, we present results under 2,000 simulation.

### 5 Results

All methods produced population-adjusted effect estimates in all but one simulated scenario. In the situation where 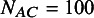 and 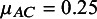 , MAIC failed to find suitable weights in 43 and 44 out 2,000 replicated datasets in the Normal and non-Normal cases, respectively. However, since G-MAIC does not exhibit any estimation problems, it seems that feasible weighting solutions are available using the full IPD, but are harder to find in bootstrapped samples. This could indicate that the proposed method is more stable than the classic MAIC.

There are also 10–20 estimations that yield treatment effect estimates of negative infinity for both of the parametric G-computation methods, regardless of the covariate structures (more in the non-Normal scenario due to the slightly more skewed distribution). This is because the predicted outcome probabilities in the 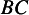 population in those datasets were exactly 1, which equals infinity after a logit transformation. This is unlikely to be an issue in practice, as these data structures are unlikely to be replicated in reality. All those problematic results were considered missing when analysing the results.

We found our findings robust to a large number of Monte Carlo replications. Here we presented the results with 2,000 Monte Carlo replications.

#### 5.1 Bias

**Figure 2:**
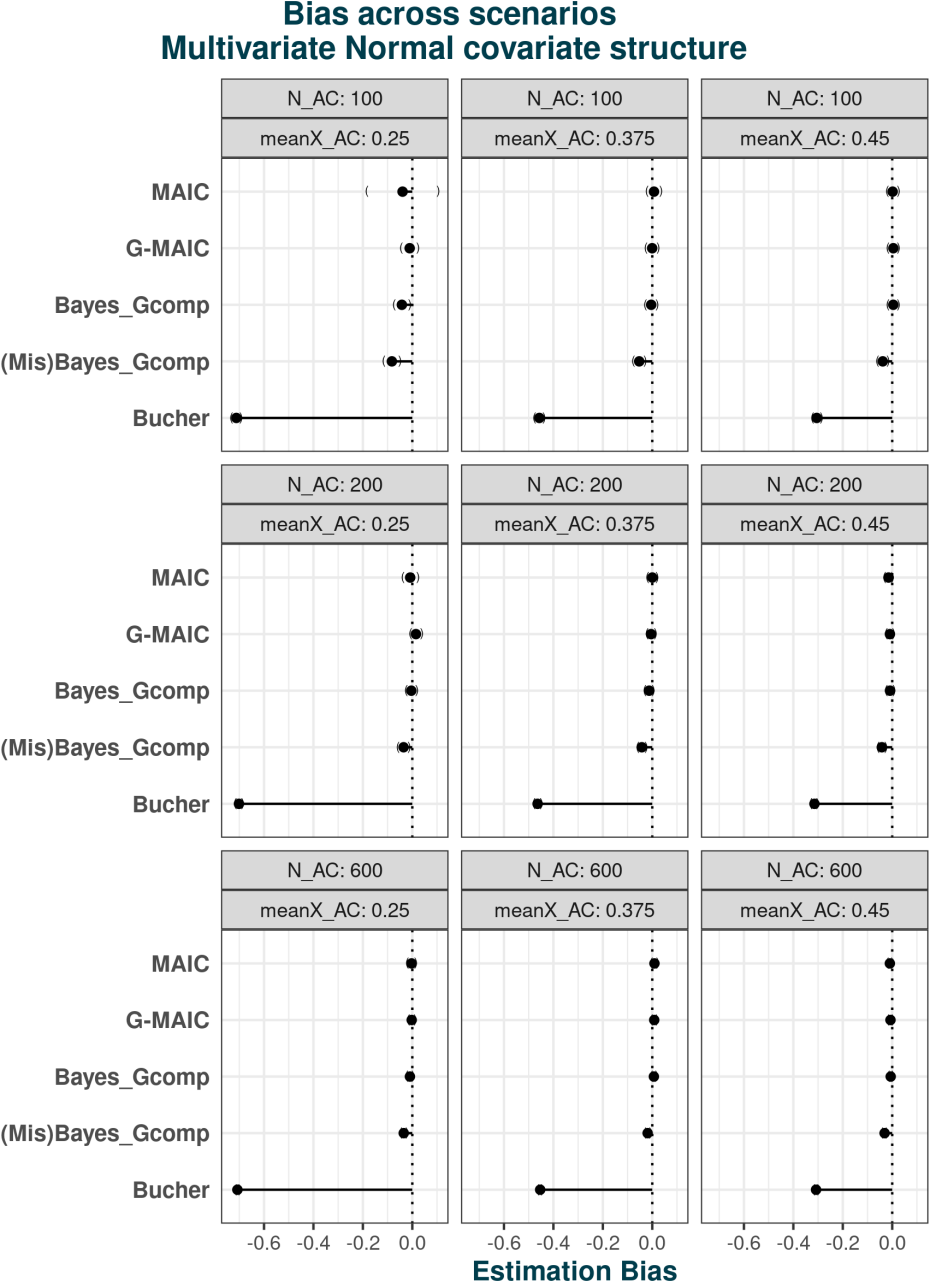
Estimation bias under multivariate Normal covariate structure with varying overlap and sample size: from left to right average sample size reductions are 82.7% 55% and 31%. *Note*: From top to bottom, methods are displayed in the order of: MAIC, G-MAIC, Bayesian Parametric G-computation, Bayesian Parametric G-computation under misspecified covariate model, Bucher’s method.

**Figure 3:**
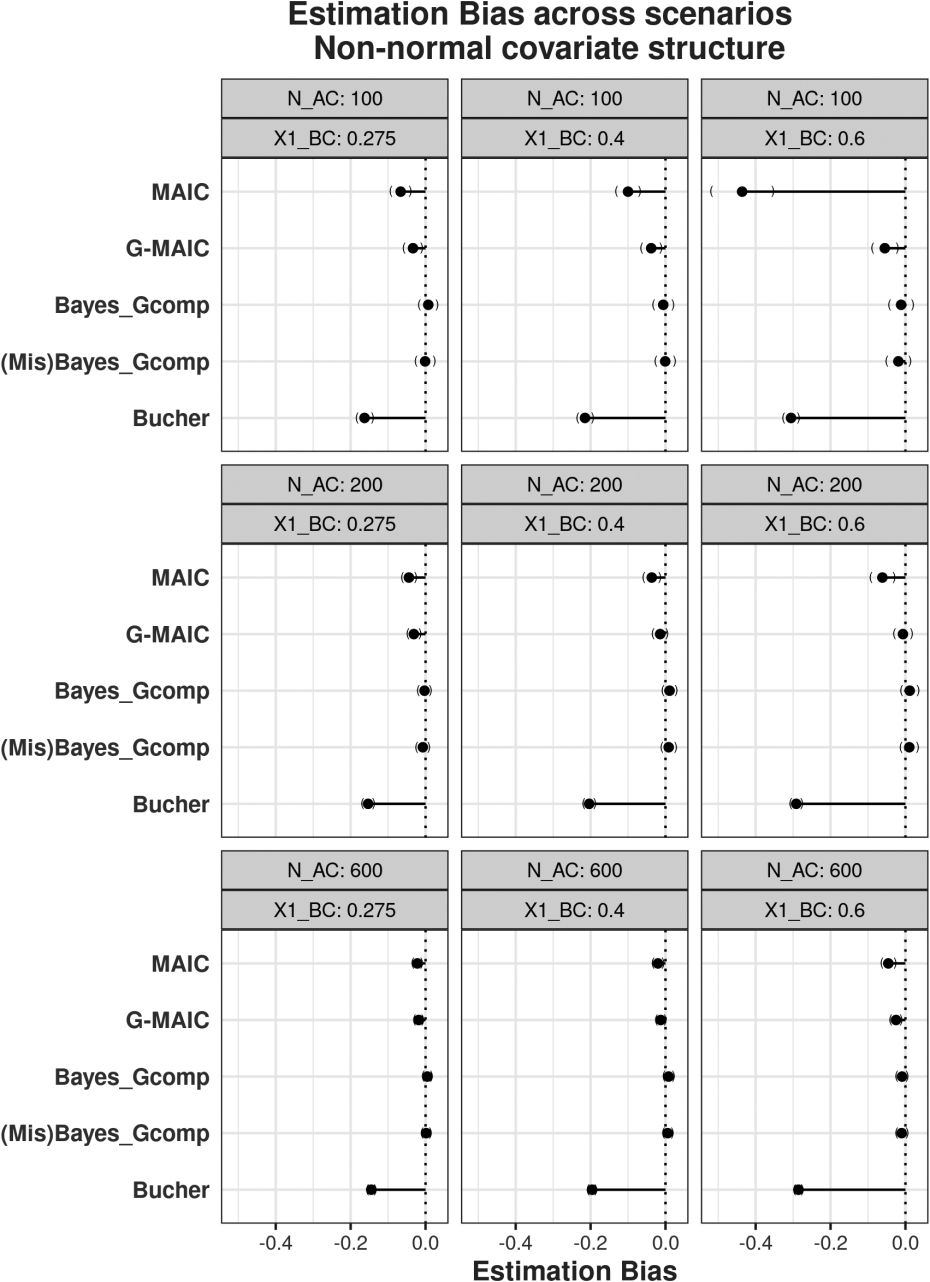
Esitmation bias under non-Normal covariate structure with varying overlap and sample size: from left to right average sample size reductions are 32.7% 55% and 81%. *Note*: From top to bottom, methods are displayed in the order of: MAIC, G-MAIC, Bayesian Parametric G-computation, Bayesian Parametric G-computation under mis-specified covariate model, Bucher’s method.

The bias across scenarios under the different covariate structures is shown in Figures 2 and 3. For normally distributed covariates, where a logistic regression model is implied for the trial assignment, MAIC estimates the population-adjusted estimate with minimal bias, even with small samples and poor overlap. However, the large MCSE indicates that the estimates are highly variable across the simulations. Our proposed G-MAIC recovers unbiased estimates across all scenarios and produces less variable estimates with smaller bias compared to MAIC in extreme scenarios. Bayesian parametric G-computation also has minimal bias under correctly parametrised covariate distributions but biased estimates under mis-specification, increasing as IPD sample size decreases.

Results change when covariates are generated under the non-normal structure. Parametric G-computation with a misspecified population remains accurate, suggesting that the multivariate normal distribution approximates the true population adequately. Nevertheless, binary covariates and non-linear dependence structure lead to the incorrect specification of implied assignment function for MAIC. As shown in 3, estimates from MAIC become biased. In the worst performing scenario—where smallest IPD sample size coincides with poor population overlap—the bias from MAIC exceeds that of unadjusted comparisons. In this scenario, the low absolute ESS, a direct result of poor overlap combined with already limited sample size, becomes further compounded by bootstrap resampling, ultimately inducing finite-sample bias within logistic regression estimates. Indeed, as illustrated by Figure 3, at similarly poor levels of overlap but with larger IPD sample sizes, the resulting bias is notably smaller. Meanwhile, the issues caused by extreme weights can be partly alleviated by a correct outcome model—G-MAIC gives estimates with much smaller bias in small samples and poorly overlapped cases, despite its slightly worse performance compared to the fully parametric methods.

#### 5.2 Variance and variance estimation

Tables 2 and 3 show that MAIC estimates’ variability is determined by the ESS. The empirical standard error of the estimator peaks under the smallest effective sample size, regardless of covariate structure. However, G-MAIC produces more stable estimates in these extreme conditions. The variability of G-MAIC is close to the fully parametric methods. This improvement becomes more prominent as unadjusted estimates become more variable. This is due to augmentation with a regression model and the use of the full samples instead of non-parameteric bootstrapped samples for variance estimation. Therefore, re-weighted populations in G-MAIC will always have larger ESS. Additionally, using weights for averaging potential outcomes can be more stable than weighted regressions. Finally, our results indicate that dependence structures among covariates do not significantly impact variability.

**Table 2:** Empirical and Model SE across scenarios under multivariate Normal DGP

Statistics	Sample size	IPD location	Bucher	Bayes Gcomp	G-MAIC	MAIC	Bayes Gcomp(mis-specified)
Poor overlap							
Empirical SE	100	0.25	0.458(0.00724)	0.744(0.0118)	0.783(0.0124)	1.913(0.0306)	0.743(0.0118)
Model SE	100	0.25	0.452(0.000316)	0.701(0.00159)	0.697(0.00189)	3.841(0.1380)	0.711(0.00144)
Empirical SE	200	0.25	0.341(0.00539)	0.512(0.00809)	0.522(0.00826)	0.71(0.0112)	0.511(0.00808)
Model SE	200	0.25	0.338(0.000133)	0.517(0.000755)	0.496(0.000849)	0.84(0.0159)	0.518(0.000713)
Empirical SE	600	0.25	0.231(0.00365)	0.313(0.00495)	0.314(0.00497)	0.409(0.00648)	0.31(0.0049)
Model SE	600	0.25	0.237(3.72e-05)	0.329(0.000259)	0.312(0.000282)	0.398(0.000848)	0.327(0.000244)
Moderate overlap							
Empirical SE	100	0.375	0.439(0.00695)	0.577(0.00913)	0.57(0.00902)	0.66(0.0104)	0.577(0.00912)
Model SE	100	0.375	0.44(0.000155)	0.58(0.00104)	0.536(0.00102)	0.719(0.0111)	0.585(0.000978)
Empirical SE	200	0.375	0.332(0.00525)	0.4(0.00633)	0.398(0.0063)	0.446(0.00705)	0.399(0.00631)
Model SE	200	0.375	0.331(6.42e-05)	0.432(0.000507)	0.395(0.000485)	0.445(0.00088)	0.433(0.000451)
Empirical SE	600	0.375	0.232(0.00366)	0.264(0.00418)	0.263(0.00416)	0.287(0.00454)	0.262(0.00414)
Model SE	600	0.375	0.234(2.31e-05)	0.285(0.000171)	0.264(0.000165)	0.286(0.000295)	0.285(0.000165)
Good overlap							
Empirical SE	100	0.45	0.434(0.00686)	0.515(0.00814)	0.501(0.00793)	0.534(0.00845)	0.518(0.0082)
Model SE	100	0.45	0.437(9.98e-05)	0.53(0.000802)	0.474(0.000685)	0.536(0.00108)	0.535(0.000734)
Empirical SE	200	0.45	0.331(0.00523)	0.37(0.00585)	0.362(0.00573)	0.38(0.006)	0.367(0.00581)
Model SE	200	0.45	0.329(3.84e-05)	0.398(0.000387)	0.354(0.000332)	0.38(0.000454)	0.399(0.000339)
Empirical SE	600	0.45	0.237(0.00375)	0.25(0.00395)	0.248(0.00392)	0.257(0.00407)	0.249(0.00394)
Model SE	600	0.45	0.233(1.93e-05)	0.268(0.000134)	0.245(0.000119)	0.254(0.000148)	0.268(0.000129)

**Table 3:** Empirical and Model SE across scenarios under non-Normal DGP

Statistics	Sample size	IPD location	Bucher	Bayes Gcomp	G-MAIC	MAIC	Bayes Gcomp(mis-specified)
Good overlap							
Empirical SE	100	0.275	0.483(0.00765)	0.545(0.00863)	0.55(0.00871)	0.603(0.00954)	0.55(0.00872)
Model SE	100	0.275	0.464(0.000452)	0.547(0.00115)	0.505(0.00094)	0.604(0.00317)	0.551(0.00121)
Empirical SE	200	0.275	0.344(0.00545)	0.384(0.00608)	0.382(0.00603)	0.408(0.00645)	0.389(0.00615)
Model SE	200	0.275	0.347(0.000176)	0.416(0.000547)	0.377(0.000443)	0.409(0.000618)	0.419(0.000557)
Empirical SE	600	0.275	0.251(0.00398)	0.268(0.00424)	0.265(0.0042)	0.276(0.00437)	0.269(0.00425)
Model SE	600	0.275	0.243(5.46e-05)	0.282(0.000181)	0.258(0.000147)	0.27(0.000199)	0.283(0.000185)
Moderate overlap							
Empirical SE	100	0.4	0.465(0.00735)	0.589(0.00933)	0.606(0.00959)	0.706(0.0112)	0.594(0.00941)
Model SE	100	0.4	0.464(0.000442)	0.594(0.00137)	0.564(0.0012)	0.812(0.0116)	0.596(0.00136)
Empirical SE	200	0.4	0.345(0.00546)	0.43(0.00679)	0.429(0.00678)	0.487(0.0077)	0.434(0.00686)
Model SE	200	0.4	0.346(0.000188)	0.45(0.000706)	0.416(0.000593)	0.48(0.00118)	0.452(0.000697)
Empirical SE	600	0.4	0.248(0.00392)	0.286(0.00452)	0.283(0.00448)	0.309(0.00489)	0.287(0.00454)
Model SE	600	0.4	0.242(5.45e-05)	0.299(0.000235)	0.278(0.000199)	0.304(0.000346)	0.3(0.000238)
Poor overlap							
Empirical SE	100	0.6	0.468(0.0074)	0.707(0.0113)	0.769(0.0122)	1.86(0.0297)	0.71(0.0113)
Model SE	100	0.6	0.464(0.000494)	0.692(0.0017)	0.699(0.00202)	3.63(0.109)	0.695(0.0018)
Empirical SE	200	0.6	0.354(0.0056)	0.512(0.0081)	0.524(0.00829)	0.724(0.0114)	0.517(0.00817)
Model SE	200	0.6	0.345(0.000188)	0.519(0.000886)	0.501(0.000817)	0.822(0.0143)	0.523(0.000896)
Empirical SE	600	0.6	0.24(0.0038)	0.33(0.00522)	0.328(0.00519)	0.418(0.00661)	0.331(0.00524)
Model SE	600	0.6	0.241(5.02e-05)	0.338(0.000311)	0.32(0.000276)	0.408(0.000877)	0.339(0.000307)

We evaluate variance estimation by comparing empirical standard errors and model average standard errors. Firstly, we confirm that the variance estimation from MAIC can be unstable under poor population overlap. Comparatively speaking, variance estimation for G-MAIC is much more stable but tends to underestimate true variability— especially under small ESS—due to concentration of weights leading to an overly homogeneous ALD approximation. Fully parametric methods perform well as they extrapolate and consider the variance information when simulating the ALD.

#### 5.3 Coverage

**Figure 4:**
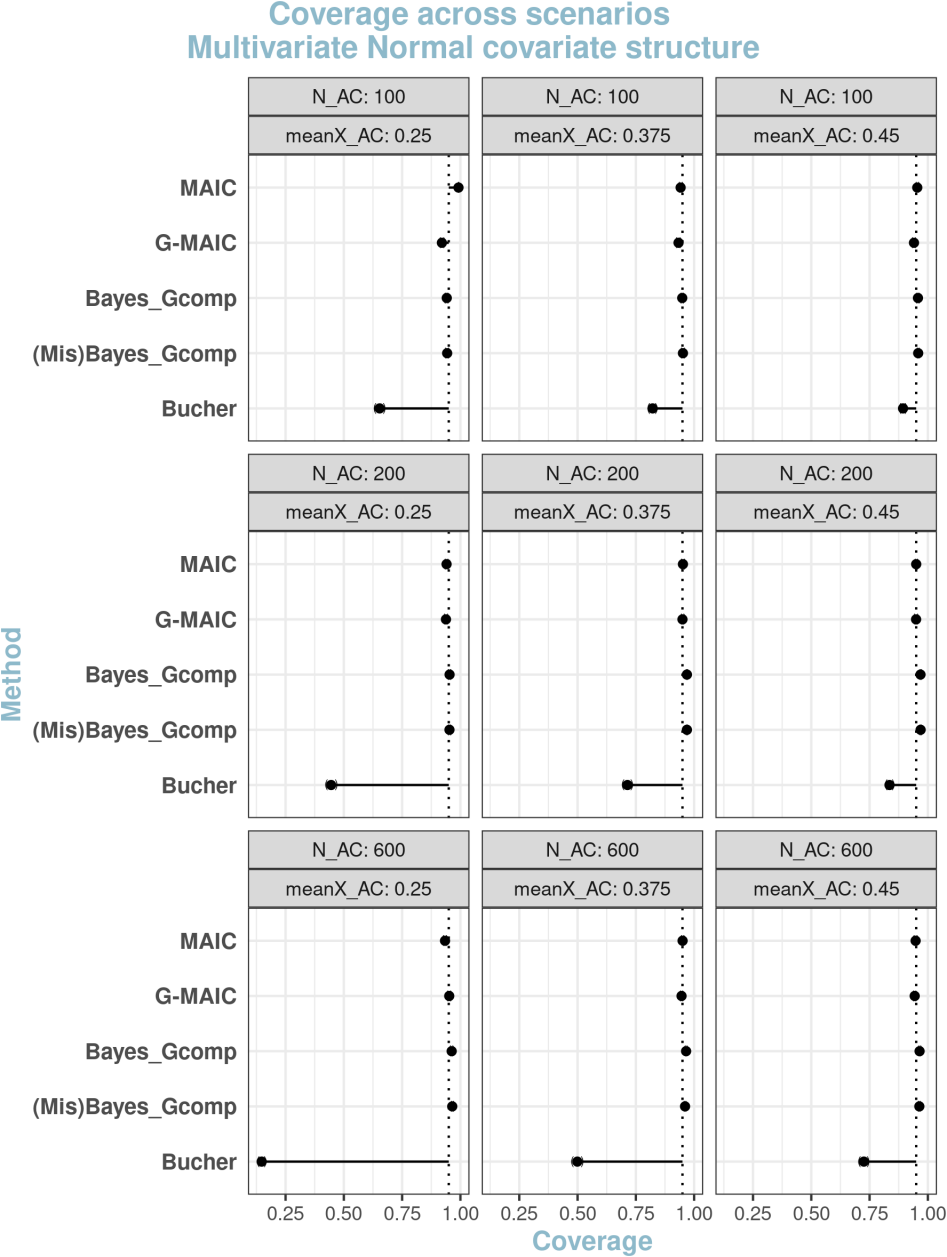
Coverage of 95% confidence intervals under multivariate Normal covariate structure with varying overlap and sample size: from left to right average sample size reductions are 82.7% 55% and 31%. *Note*: From top to bottom, methods are displayed in the order of: MAIC, G-MAIC, Bayesian Parametric G-computation, Bayesian Parametric G-computation under misspecified covariate model, Bucher’s method.

**Figure 5:**
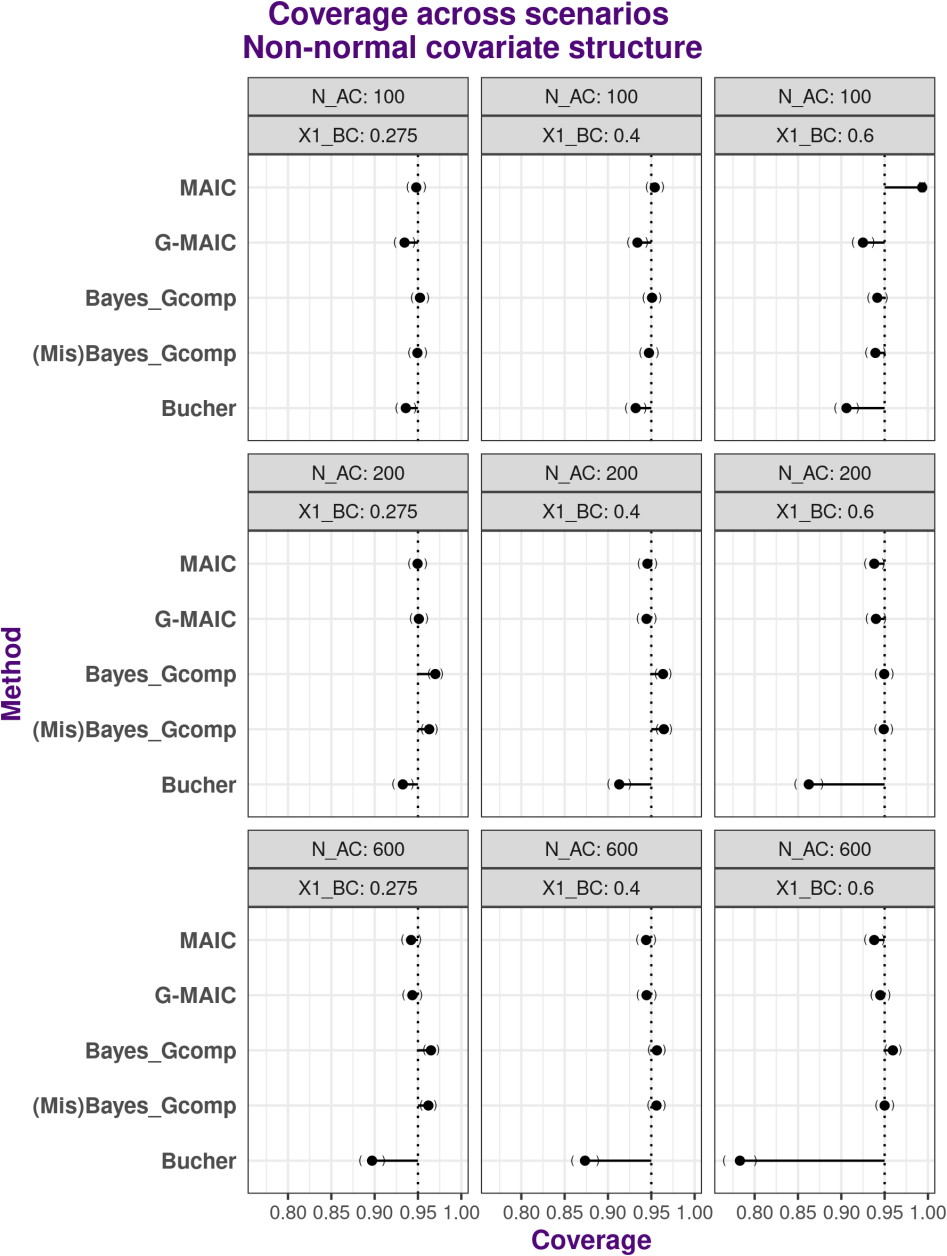
Coverage of 95% confidence intervals under non-Normal covariate structure with varying overlap and sample size: from left to right average sample size reductions are 32.7% 55% and 81%. *Note*: From top to bottom, methods are displayed in the order of: MAIC, G-MAIC, Bayesian Parametric G-computation, Bayesian Parametric G-computation under mis-specified covariate model,Bucher’s method.

Figures 4 and 5 display the coverage of the 95% confidence intervals for normal and non-normal covariate structures, respectively. MAIC exhibits over-coverage in extreme scenarios due to failed variance estimation. In less extreme cases, the Normal covariate structure exhibits largely correct coverage due to minimal bias and largely correct variance estimation. Conversely, under the non-normal covariate structure, the large estimation bias can sometimes ‘over-compensate’ exaggerated model variance leading to correct coverage.

G-MAIC shows some under-coverage mainly driven by an underestimation of true variance; thus, nominal 95% confidence interval coverage is worst with small ESS values. For fully parametric methods, the parameterically specified populations offers sufficiently good approximations to the underlying ALD covariate structure, so the coverage is close to the nominal 95%.

### 6 Discussion

In the real-world, regulators face increasing amounts of efficacy evidence that are based on small trials that do not compare all relevant interventions. This is especially true for interventions for rare and more severe diseases. In these scenarios, it is valuable to conduct population adjustment to enable ‘fairer’ comparisons between the available interventions. However, our simulation study demonstrates that even when the trial assignment model is correctly specified, the large variability along with unstable variance estimation, could render the standard MAIC method almost unusable. At the same time, the regulator might be reluctant to accept results from parametric G-computation since it is based on simulated covariates. Thus, we introduced G-MAIC that combines outcome regression with the weighting adjustment used in MAIC. We have shown in a comprehensive simulation study that it achieves better performance in terms of both unbiasedness and stable variance estimation that MAIC and that it is more robust under mis-specification of the trial assignment model.

The proposed G-MAIC method can offer an acceptable compromise between MAIC and parametric G-computation. With the correct trial assignment weights, G-MAIC provides an adjusted estimate with minimal bias and stable variance estimation. Apart from the efficiency gains due to the usage of a outcome model, the improved performance can also be explained by the use of Bayesian bootstrap, which provides a more stable variance estimate. G-MAIC does underestimate variance in small samples and warrants further investigations. ESS after estimating the weights can diagnose issues just as in MAIC. Analysts adopting G-MAIC could consider inflating the variance by slightly tempering the estimated weights when faced with extremely small ESS (raise the weights to a power that is smaller than 1). However, future work could focus on developing a more principled way to incorporate the uncertainty in weights estimation.

Furthermore, a bigger challenge for MAIC is the mis-specification of the trial assignment model. Under such mis-specification, the re-weighted population is no longer exchangeable with the ALD population, and mean-balancing alone is insufficient to correct all the bias from effect modifications in non-linear models. In HTA, effect-modifiers typically come in different data types, which implies that the trial assignment models can often be mis-specified. The simulation results under the non-normal data structure indicate issues with the use of MAIC in practice. We observe that low absolute ESS can exacerbate the problem non-exchangeability. Under the most extreme scenario, where the absolute ESS is drastically reduced due to the compounded effect of poor overlap and small IPD sample sizes, MAIC can produce estimates that are more biased than the unadjusted method. Previous simulation studies have assumed a multivariate Normal covariate structure, this is one of the few studies that explores how non-Normal covariate structure affects the MAIC estimates.

G-MAIC on the other hand, substantially improves over MAIC in our simulations despite using the same weights for re-weighting. This is likely due to a correctly specified outcome model. The estimate under mis-specified weights is akin to the ‘true’ treatment effect in a population that is not exchangeable with the ALD. And therefore the resulting bias depends upon the degree of non-exchangeability. It is not unimaginable that the improvements can disappear in situations where the trial populations have completely different covariate structure with different skewness. The non-exchangeability is not severe in the scenarios we tested on its own but could worsen as ESS decrease. As seen in Figure 3, the estimation bias is smaller in less extreme scenarios. In scenarios where the IPD sizes are 200 and 600, G-MAIC still offers performance gains, but to smaller extent as the ESS becomes sufficiently large.

Nevertheless, we hesitate to conclude that G-MAIC offers a perfect solution to the problem of mis-specified assignment model, as the estimand in these cases is not so interpretable. Strictly from an estimand perspective, marginalising the correct outcome relationship in a non-exchangeable population might be regarded as irrelevant to the indirect comparison problem at hand. However, we argue that all population adjustment methods suffer from this problem—all estimands are irrelevant when the covariate model is mis-specified. On a more practical level, when faced with covariate structures with potentially different skewness, one should consider adopting alternative weighting schemes that correspond to a more appropriate trial assignment model, or even matching on covariate medians in addition to the original formulation of MAIC. The connections between calibration weights and covariate structure are briefly mentioned in,^19^ ^,^ ^32^ and is an area for future work.

In addition, methods such as MAIC, parametric G-computation, and the proposed G-MAIC inherently produce treatment effect estimates anchored exclusively to the specific ALD trial population, substantially limiting their synthesis into larger evidence networks. As evidence accumulates and the assumption of shared effect modifiers becomes increasingly justifiable, ML-NMR emerges as a desirable alternative, as it integrates evidence from multiple IPD and ALD studies and enables the estimation of marginal or conditional effects relevant to any selected decision-making target population of interest. Nonetheless, the increased complexity, data requirements, and the stronger assumptions around shared effect-modifiers may present practical challenges. Thus, methods only applicable to pairwise indirect comparisons remain relevant in settings with early-stage evidence.

Recent work by Park et al.^40^ has highlighted the growing interest in doubly-robust methods for population-adjusted indirect comparisons, which ensures the consistency of the estimator when either the outcome or assignment model is correctly specified. Their approach combines MAIC weights with an outcome regression model for unanchored comparisons involving survival outcomes. While the research in doubly-robust methods continue to evolve, the G-MAIC offers a flexible framework to accommodate all possible methods: the central goal of G-MAIC is to use Bayesian bootstrap to construct the ALD population non-parametrically, while any suitable outcome modelling approach can be applied in the first-stage regression.

In conclusion, we emphasise two critical aspects of population adjustment: target population and extrapolation methods. G-MAIC adopts MAIC weights, rendering estimates a ‘targeted comparison’ within the ALD trial population. This ‘fixation’ on the target population can yield different estimates for the same trial pairs, contingent on ALD/IPD availability. However, this is not a major limitation, as marginal estimands are sample-specific under effect-modifications. For G-MAIC, the target population is an interpolation between weighted and unweighted IPD. Although differ from the decision population, it might be the best compromise without additional parametric assumptions.

Extrapolation considerations are complex. G-MAIC does not extend beyond the IPD sample space, making it sensitive to population overlap but enabling the safe adoption of novel Bayesian non-parametric methods, such as Bayesian additive regression trees for outcome modelling. Extrapolations from these methods can be unreliable, relying on limited observations at the IPD covariate boundaries. In summary, future research should focus on innovative approaches to reconstruct the target population based on ALD and develop methods for robust non-linear extrapolations.

## Data Availability

Data availability is not applicable to this article as no new data were created or analysed in this study.

## Footnotes

1 Alternative sets of weights that enforce balance of covariate means across trials could be used instead.

## Appendix

- The number of participants in the IPD trial,  . We will use a  allocation ratio for both sample sizes;
- The covariate structures of IPD in both trials:
  1. The Normal case: five continuous covariates with each covariate *k* in trial j generated from a Normal distribution:  . The pairwise correlation  has that
  2. The non-Normal case: starting with a Normally distributed covariate  , a binary covariate  is generated such that  . The third covariate  depend on  with  .  is generated from a Gamma distribution with mean  , and dispersion parameter  . Finally, a binary  is generated with  .
- The overlap between trials: overlap between multivariate distributions is complex and hard to compute. Instead, we choose the parameter values for the distributions to directly target a corresponding reduction in ESS. This is achieved by repeatedly applying MAIC to 2,000 simulated  and  populations with  , and compute the average reduction ESS across all repetitions. In the Normal case, we have  . The population standard deviation is fixed to be  for both trials. The marginal mean in the  population is set at  . corresponding to 31%, 54% and 82.7% reduction in ESS. In the second scenario, we set  and  , corresponding to  and  reduction in ESS.

## References

[1] World Health Organization, Regional Office for Europe, Health Evidence Network, European Observatory on Health Systems and Policies, Sorenson C , Drummond M , Kristensen FB , et al. How can the impact of health technology assessments be enhanced? World Health Organization, Regional Office for Europe; 2008. https://iris.who.int/handle/10665/107981.

[2] O’Rourke B , Oortwijn W , Schuller T . Announcing the new definition of health technology assessment. Value Health 2020;23(6):824–825. 32540240 10.1016/j.jval.2020.05.001

[3] Guyatt G , Oxman AD , Akl EA , et al. Grade guidelines: 1. Introduction—Grade evidence profiles and summary of findings tables. J. Clin. Epidemiol. 2011;64(4):383–394. 21195583 10.1016/j.jclinepi.2010.04.026

[4] Sorenson C , Drummond M , Kristensen FB , et al. How Clinicanl the Impact of Health Technology Assessments be Enhanced? Citeseer John Wiley & Sons; 2008.

[5] Bucher HC , Guyatt GH , Griffith LE , Walter SD . The results of direct and indirect treatment comparisons in meta-analysis of randomized controlled trials. J. Clin. Epidemiol. 1997;50(6):683–691. 9250266 10.1016/s0895-4356(97)00049-8

[6] Dias S , Sutton AJ , Ades A , Welton NJ . Evidence synthesis for decision making 2: A generalized linear modeling framework for pairwise and network meta-analysis of randomized controlled trials. Med. Decision Making 2013;33(5):607–617. 10.1177/0272989X12458724 PMC3704203 23104435

[7] Westreich D , Edwards JK , Lesko CR , Stuart E , Cole SR . Transportability of trial results using inverse odds of sampling weights. Amer. J. Epidemiol. 2017;186(8):1010–1014. 28535275 10.1093/aje/kwx164 PMC5860052

[8] Tremblay G , Chandiwana D , Dolph M , Hearnden J , Forsythe A , Monaco M . Matching-adjusted indirect treatment comparison of ribociclib and palbociclib in HR+, HER2- advanced breast cancer. Cancer Manag. Res. 2018;10:1319–1327. 10.2147/CMAR.S163478. 29861642 PMC5968783

[9] Truong B , Tran LAT , Le TA , Pham TT , Vo TT . Population adjusted-indirect comparisons in health technology assessment: A methodological systematic review. Res. Synth. Methods 2023;14(5):660–670. 37400080 10.1002/jrsm.1653

[10] Kalf R , Dawoud D , Bregman C , et al. Pp146 the use of indirect comparisons for reimbursement decision making in the Netherlands and England. Int. J. Technol. Assess. Health Care 2022;38(S1):S87–S87.

[11] Signorovitch JE , Wu EQ , Yu AP , et al. Comparative effectiveness without head-to-head trials: A method for matching-adjusted indirect comparisons applied to psoriasis treatment with adalimumab or etanercept. Pharmacoeconomics 2010;28:935–945. 20831302 10.2165/11538370-000000000-00000

[12] Signorovitch JE , Sikirica V , Erder MH , et al. Matching-adjusted indirect comparisons: A new tool for timely comparative effectiveness research. Value Health 2012;15(6):940–947. 22999145 10.1016/j.jval.2012.05.004

[13] Ishak K , Rael M , Phatak H , et al. Simulated treatment comparison of time-to-event (and other non-linear) outcomes. Value Health 2015;18(7):A719.

[14] Remiro-Azócar A , Heath A , Baio G . Parametric g-computation for compatible indirect treatment comparisons with limited individual patient data. Res. Synth. Methods 2022;13(6):716–744. 35485582 10.1002/jrsm.1565 PMC9790405

[15] Phillippo DM , Dias S , Ades A , et al. Multilevel network meta-regression for population-adjusted treatment comparisons. J. Royal Stat. Soc. Ser. A: Stat. Soc. 2020;183(3):1189–1210. 10.1111/rssa.12579 PMC7362893 32684669

[16] Phillippo D , Ades T , Dias S , Palmer S , Abrams KR , Welton N . NICE DSU technical support document 18: Methods for population-adjusted indirect comparisons in submissions to NICE. Decision Support Unit, ScHARR, University of Sheffield: NICE Decision Support Unit; 2016. 81 pp (Technical Support Documents).

[17] Phillippo DM , Ades AE , Dias S , Palmer S , Abrams KR , Welton NJ . Methods for population-adjusted indirect comparisons in health technology appraisal. Med. Decision Making 2018;38(2):200–211. 10.1177/0272989X17725740 PMC5774635 28823204

[18] Remiro-Azócar A , Heath A , Baio G . Methods for population adjustment with limited access to individual patient data: A review and simulation study. Res. Synth. Methods 2021;12(6):750–775. 34196111 10.1002/jrsm.1511

[19] Wang J. On matching-adjusted indirect comparison and calibration estimation. 2021. http://arxiv.org/abs/2107.11687.

[20] Phillippo DM , Dias S , Ades AE , Welton NJ . Assessing the performance of population adjustment methods for anchored indirect comparisons: A simulation study. Stat. Med. 2020;39(30):4885–4911. 10.1002/sim.8759. 33015906 PMC8690023

[21] Efron B. Nonparametric estimates of standard error: the jackknife, the bootstrap and other methods. Biometrika 1981;68(3):589–599.

[22] Phillippo DM . Calibration of Treatment Effects in Network Meta-Analysis Using Individual Patient Data. PhD Thesis. University of Bristol; 2019.

[23] Ishak KJ , Proskorovsky I , Benedict A . Simulation and matching-based approaches for indirect comparison of treatments. Pharmacoeconomics 2015;33(6):537–549. 10.1007/s40273-015-0271-1. 25795232

[24] Remiro-Azócar A. Target estimands for population-adjusted indirect comparisons. Stat. Med. 2022;41(28):5558–5569. 10.1002/sim.9413. 36385476

[25] Daniel R , Zhang J , Farewell D . Making apples from oranges: Comparing noncollapsible effect estimators and their standard errors after adjustment for different covariate sets. Biom. J. 2021;63(3):528–557. 33314251 10.1002/bimj.201900297 PMC7986756

[26] Phillippo DM , Dias S , Ades AE , Welton NJ . Target estimands for efficient decision making: Response to comments on “assessing the performance of population adjustment methods for anchored indirect comparisons: A simulation study”. Stat. Med. 2021;40(11):2759. 33963586 10.1002/sim.8965 PMC9495275

[27] Remiro-Azócar A , Heath A , Baio G . Model-based standardization using multiple imputation. *BMC Med. Res. Methodol.* 2024;24:32. 10.1186/s12874-024-02157-x. PMC10858574 38341552

[28] Rubin DB . The Bayesian bootstrap. Ann. Stat. 1981;9:130–134. 10.1214/aos/1176345338.

[29] Nie L , Soon G . A covariate-adjustment regression model approach to noninferiority margin definition. Stat. Med. 2010;29(10):1107–1113. 20209669 10.1002/sim.3871

[30] Nie L , Zhang Z , Rubin D , Chu J . Likelihood reweighting methods to reduce potential bias in noninferiority trials which rely on historical data to make inference. Ann. Appl. Stat. 2013;7:1796–1813.

[31] Josey KP , Berkowitz SA , Ghosh D , Raghavan S . Transporting experimental results with entropy balancing. Stat. Med. 2021;40(19):4310–4326. 10.1002/sim.9031. 34018204 PMC8487904

[32] Jackson D , Rhodes K , Ouwens M . Alternative weighting schemes when performing matching-adjusted indirect comparisons. Res. Synth. Methods 2021;12(3):333–346. 10.1002/jrsm.1466. 33131206

[33] Oganisian A , Roy JA . A practical introduction to Bayesian estimation of causal effects: Parametric and nonparametric approaches. Stat Med 2021;40(2): 518–551. 10.1002/sim.8761. 33015870 PMC8640942

[34] Malya G , Glen M . Bayesian Methods for Finite Population Sampling. Chapman and Hall; 1997. 10.1201/9781315138169.

[35] Lo AY . A Bayesian bootstrap for a finite population. Ann. Stat. 1988;16:1684–1695. 10.1214/aos/1176351061.

[36] Fong E , Holmes C , Walker SG . Martingale posterior distributions. *J. Royal Stat. Soc. B: Stat. Methodol.* 2023;85(5):1357–1391. 10.1093/jrsssb/qkad005.

[37] Morris TP , White IR , Crowther MJ . Using simulation studies to evaluate statistical methods. Stat. Med. 2019;38(11):2074–2102. 30652356 10.1002/sim.8086 PMC6492164

[38] R Core Team. R: A Language and Environment for Statistical Computing. R Foundation for Statistical Computing; 2021. https://www.R-project.org/.

[39] Stanley K. Design of randomized controlled trials. Circulation 2007;115(9):1164–1169. 17339574 10.1161/CIRCULATIONAHA.105.594945

[40] Park JE , Campbell H , Towle K , et al. Unanchored population-adjusted indirect comparison methods for time-to-event outcomes using inverse odds weighting, regression adjustment, and doubly robust methods with either individual patient or aggregate data. Value Health 2024;27(3):278–286. 38135212 10.1016/j.jval.2023.11.011

